# New Bacterial Phytase through Metagenomic Prospection

**DOI:** 10.3390/molecules23020448

**Published:** 2018-02-17

**Authors:** Nathálya Farias, Isabela Almeida, Carlos Meneses

**Affiliations:** 1 Graduate Program in Agricultural Sciences, Universidade Estadual da Paraíba (UEPB), Campina Grande/PB 58429-500, Brazil; nathalyacfarias@gmail.com; 2 Department of Biology, Universidade Estadual da Paraíba (UEPB), Campina Grande/PB 58429-500, Brazil; isabelaalmeida42@outlook.com; 3 Department of Biology and Graduate Program in Agricultural Sciences, Universidade Estadual da Paraíba (UEPB), Campina Grande/PB 58429-500, Brazil

**Keywords:** crop residues, functional metagenomics, phytasic activity

## Abstract

Alkaline phytases from uncultured microorganisms, which hydrolyze phytate to less phosphorylated myo-inositols and inorganic phosphate, have great potential as additives in agricultural industry. The development of metagenomics has stemmed from the ineluctable evidence that as-yet-uncultured microorganisms represent the vast majority of organisms in most environments on earth. In this study, a gene encoding a phytase was cloned from red rice crop residues and castor bean cake using a metagenomics strategy. The amino acid identity between this gene and its closest published counterparts is lower than 60%. The phytase was named PhyRC001 and was biochemically characterized. This recombinant protein showed activity on sodium phytate, indicating that PhyRC001 is a hydrolase enzyme. The enzymatic activity was optimal at a pH of 7.0 and at a temperature of 35 °C. β-propeller phytases possess great potential as feed additives because they are the only type of phytase with high activity at neutral pH. Therefore, to explore and exploit the underlying mechanism for β-propeller phytase functions could be of great benefit to biotechnology.

## 1. Introduction

The nutritional requirements of plants, considering a mineral balance that provides the maximum performance at lower cost, have been examined in recent studies [1]. Among minerals, phosphorus (P) stands out for its participation in many functions during plant development and its high cost in phosphorus fertilization supplementation [1,2]. The lack of this mineral in the soil is due to the fact that it has limited diffusion in most soils, where its ions are highly reactive with numerous soil constituents [3] and the plants use this nutrient almost exclusively in the form of phosphate anions, mainly HPO_4_^−2^ and H_2_PO_4_^−1^ [4].

Organic phosphorus (Po) represents up to 80% of the total P present in soils, 50% of which occurs in the form of phytate (Na-IHP). This form of Po appears to be used only slightly by plants [5]. Phytate hydrolysis is mediated specifically by phytase, whose contribution to plant nutrition has been poorly explored. It is known that the environment presents a high level of microbial biological diversity; however, only a small part of this is known [6]. Some of these microorganisms are responsible for directing the main biogeochemical cycles and therefore perform ecological functions that make them indispensable, for example through their participation in phytate hydrolysis [2].

With the development of pure culture techniques, microorganisms could be studied individually and characterized, mainly, based on nutritional criteria. However, the use of that approach limits the taxonomic and phylogenetic evaluations of some organisms, since the cultivated ones represent only a small fraction of the diversity of species in microbial communities [7]. From this perspective, metagenomics appears as the best prospecting tool to revolutionize the field of biotechnology, allowing high resolution description of complex bacterial communities in their natural environments [8,9].

Bacteria and fungi are the main sources of phytases for biotechnology [1], and most of the earth’s microorganisms are not yet cultivable using traditional techniques [10]. Therefore, isolating genes from uncultured microorganisms is an attractive target for functional metagenomics [11]. This approach has already been used to clone and characterize phosphatases and phytases [12]. However, there is still a great diversity of microbial phytases unexplored in many environments and species that have not been studied. 

While rice is a great source of soil carbon, castor bean is an organic fertilizer rich in nitrogen that has been widely used in agriculture and has great potential to provide raw material for biodiesel production [13,14]. This product originates from castor oil obtained from the castor oil plant, showing a gradual release of nutritional compounds, aiding in soil improvement [14]. A metagenomic approach to these decomposing materials seems to be a great opportunity to explore a microenvironment that is favorable for the genetic diversity of microorganisms and more likely to contain genes encoding phytases.

In this study, we applied functional metagenomics as a tool to identify a gene that encodes an enzyme with phytasic activity present in red rice and composted castor bean cake residues. The process involved the extraction of DNA from red rice residues and composted castor bean cake, the construction of a metagenomic library and the selection of clones capable of degrading phytate in agar plates. We identified a phytase, PhytRC001, which has similarity to other phytases of uncultured bacteria. This enzyme has been shown to be stable at a variety of temperatures and pHs, and represents a significant advance for the biotechnological degradation of phosphorus for mineral nutrition in plants.

## 2. Results

### 2.1. Construction and Screening of Environmental Genomic Libraries

**Figure 1:**
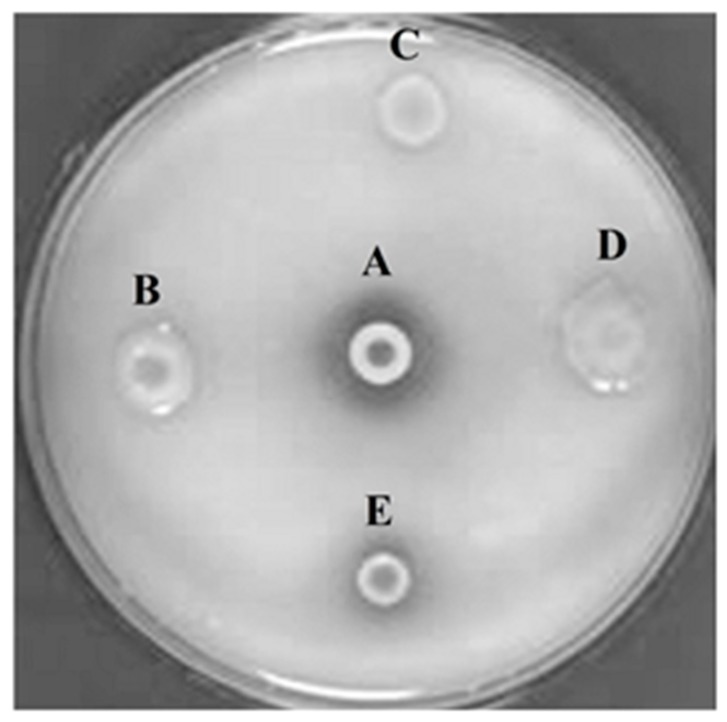
Screening for phytasic activity in metagenomic library using Na-IHP as substrate. A: positive control (*Bacillus subtilis*); B, C and D: clones with negative phytase activity; E: clone with positive phytase activity.

After extraction and purification of metagenomic DNA isolated from the cultural remains of red rice and castor bean cake, a cosmid library containing 50,000 cosmid clones (PhyRC library) was constructed from one nanogram of metagenomic DNA. A cleavage with the restriction endonuclease *BamH*I in 50 randomly chosen clones of PhyRC revealed that the clones contained DNA inserts with sizes between 20 and 60 kb; the mean size of the fragments was approximately 30 kb. A cosmid clone expressing the activity of degrading Na-IHP was isolated, after all clones from the PhyRC library were analyzed for phytase activity (Figure 1). We named this gene from cultural remains of red rice and castor cake Phytase 1 (PhyRC001) (deposited as MG544855).

### 2.2. Sequence Analyzes of the Cloned Phytase Gene

**Figure 2:**
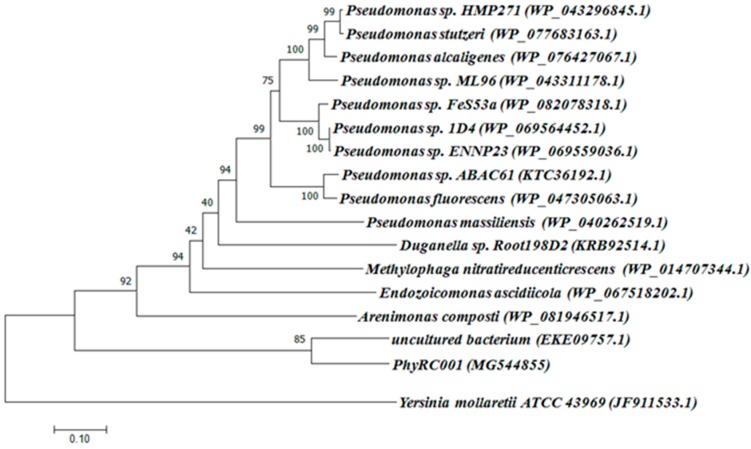
Classification of PhyRC001 based on amino acid sequence analyses. Amino acid sequences of phytases, including PhyRC001, were compared and analyzed phylogenetically using a neighbor-joining method. GenBank accession numbers are in parentheses. Phylogenetic analysis showed that PhyRC001 is closely related to phytases from an uncultured species. The histidine acid phosphatases (HAPs) phytase of *Yersinia mollaretii* ATCC 43969 (JF911533.1) was included as an outgroup.

The complete metagenomic DNA sequence inserted into the cosmid vector was determined completely by sequencing the PhyRC001 fragment. Alignment analyses, performed with the help of BLAST and ORF finder, revealed the presence of a 1203 bp open reading frame encoding a phytase (PhyRC001). The sequence for PhyRC001 (MG544855) encodes a predicted protein of 400 amino acids with a predicted molecular mass of 45.12 kDa. The deduced amino acid sequence of PhyRC001 was used for a BlastP analysis at NCBI, pFAM, SignallP 4.0 and SwissProt databases. This search revealed that PhyRC001 belonged to the β-propeller phytase family (beta-sheet motif, the enzyme’s active site is often found in the cleft formed in the center of the propeller by loops connecting the successive four-sheet motifs) and the amino acid of the gene separately shared 60% with the phytase (uncultured bacterium–EKE09757.1), and 28% with the phytase from *3-*phytase (*Arenimonas composti*–WP_081946517.1). The phylogenetic tree based on amino acid sequence was constructed to verify the evolutionary relationship of the PhyRC001 to other known phytases, and 15 phytase proteins including 10 from *Pseudomonas* were selected for the phylogenetic tree analysis. PhyRC001 is not closely related to other members of the phytase family, suggesting that it is a new member of phytase (Figure 2).

### 2.3. Expression and Purification of the Recombinant PhyRC001

**Figure 3:**
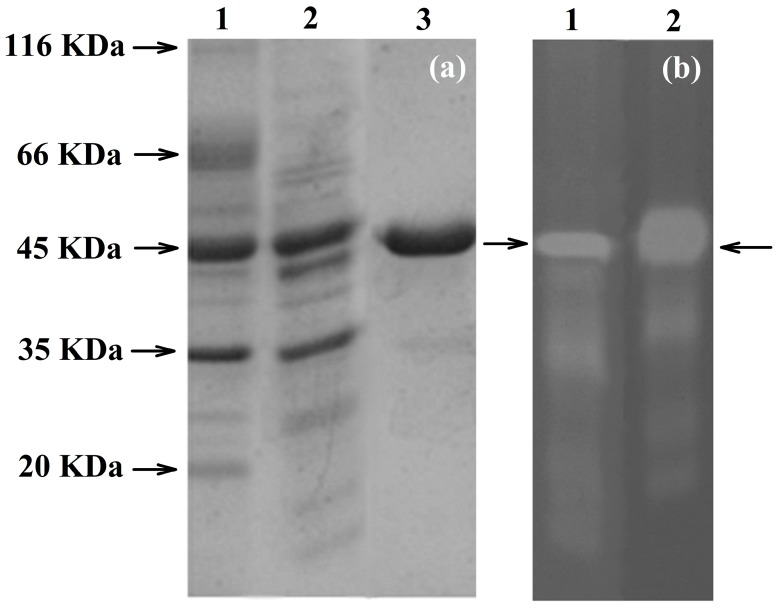
Electrophoretic analyses of PhyRC001 phytase purified from red rice crop residues and castor bean cake. (a) SDS-PAGE. 1: Molecular weight marker (kDa); 2: spin column portion of partly purified phytase (crude extract); 3: purified phytase; and (b) zymogram analysis of PhyRC001 phytase: 1: crude extract showing opaque region in native gel (arrow); 2: purified phytase showing opaque region in native gel (arrow).

To confirm the identity of PhyRC001, we purified the recombinant protein and performed assays to detect its phytase activity. The recombinant protein was purified and in vitro tests were conducted using Na-IHP zymograms (native-PAGE) to observe Na-IHPase activity. For SDS-PAGE analysis, the enzyme approximate molecular weight was estimated to be 45 kDa (Figure Figure 3a). The purified recombinant PhyRC001 protein (one microgram) was clearly active (Figure 3b). Native-PAGE and SDS-PAGE gels were used for the qualitative characterization of phytase activity. For Native-PAGE, the zymogram (0.1% Na-IHP in the gel) showed a translucent zone, indicating phytasic activity.

When PhyRC001 was subjected to Na-IHP zymogram, the degradation with a drag to the smaller molecular weight mass region was revealed, providing a strong indication that PhyRC001 may be formed by smaller protein subunits.

### 2.4. Biochemical Characterization of PhyRC001

#### 2.4.1. Temperature and pH Effect on Activity of PhyRC001

**Figure 4:**
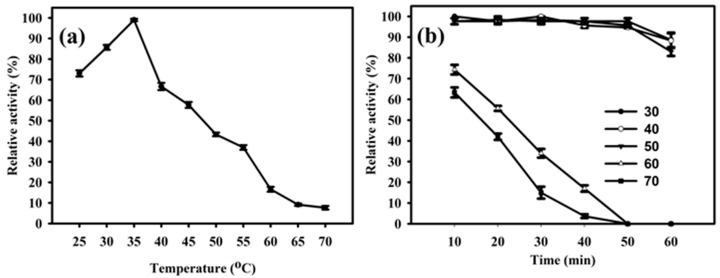
Effect of temperature on the activity and stability of PhyRC001. (**a**) Optimal temperature for PhyRC001 is 35 °C, as determined by measuring its enzymatic activity with 1% (*w*/*v*) Na-IHP in 50 mM sodium acetate buffer, pH 7, at 25 to 70 °C in five degree increments; and (**b**) thermostability was determined by measuring the enzymatic activity of PhyRC001 after incubation at 30 to 70 °C in 10 degree increments for 60 min.

The enzyme PhyRC001 showed its principal activity at temperatures between 25 to 70 °C, and the maximum activity of PhyRC001 was detected when it was incubated at 35 °C (Figure 4A). When the temperature was above 35 °C, the enzymatic activity was rapidly lost. After one hour of incubation at different temperatures, PhyRC001 retained its activity at 60 and 70 °C (Figure 4B). Cold-active enzymes are attractive because of their value in biotech applications. They are also useful tools for protein folding studies because of their high activity and stability at low temperatures [15].

**Figure 5:**
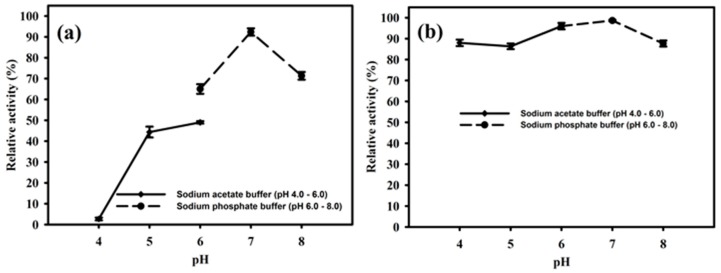
Effect of pH on the activity and stability of PhyRC001. (**a**) The optimal pH for PhyRC001 was determined by measuring the enzyme activity on 1% (*w*/*v*) Na-IHP in 50 mM buffers at 35 °C with various pH values. The buffers used to establish the optimum pH and to assess pH stability were as follows: sodium acetate buffer (pH 4–6, ♦), and sodium phosphate buffer (pH 6–8, ●); and (**b**) to determine the pH stability of PhyRC001, the enzyme was incubated for 16 h at 4 °C in buffers of different pH values. The residual enzyme activity was measured under standard assay procedures. All measurements were carried out in triplicate.

The purified enzyme PhyRC001 was active at different pH values between 4.0 and 8.0. The optimal pH of the enzyme was 7.0, where it reached a maximal enzymatic activity in this condition (Figure 5A). The enzyme activity remained high when the pH ranged from 4.0 to 6.0. However, when the pH was raised to 8.0 the activity of PhyRC001 was completely lost. After 16 h incubation at 4 °C, PhyRC001 maintained its constant activity in the pH range 4.0–6.0 (Figure 5B). The pH range of the recombinant enzyme was consistent with the alkaline phytase property reported previously [16].

#### 2.4.2. Growth, Specificity and Inhibition on Activity of PhyRC001

To confirm that the product generated by PhyRC001 possesses enzymatic phytasic activity, an assay previously described by Yanke et al. [17] was performed to monitor the release of inorganic phosphate from a phytate extract by the purified protein, at a temperature of 45 °C. The assay indicated that the purified PhyRC001 protein showed a high phytasic activity based on the rate of release of inorganic phosphate (251 ± 22 μmol P/min/g). This indication revealed that the phytase activity in PhyRC001 is sharp.

The highly specific activity of PhyRC001 (Table 1) on phytic acid, and the limited activities on *para*-Nitrophenyl phosphate, glucose 1-phosphate and glucose 6-phosphate are consistent with the result of phylogenetic analysis, which actually leads to the belief that this protein is a phytase. It is interesting to note that PhyRC001 demonstrated high phytic acid-specific activity when compared to other substrates, indicating that PhyRC001 is probably a true phytase, rather than an acid phosphatase having a contingent activity for phytic acid.

The activity for each tested substrate was determined at pH 7.0, 35 °C, and is expressed as a percentage (average of three replicates) of the activity level for phytic acid. AMP: Adenosine monophosphate; ADP: Adenosine diphosphate; ATP: Adenosine triphosphate; GTP: Guanosine-5′-triphosphate; NADP: Nicotinamide adenine dinucleotide phosphate.

Phytase activity was also verified as a function of the presence of metal ions in the reaction media for the PhyRC001 activity, which was defined with 100% activity. The results showed that the metal ions evaluated are able to inhibit phytase activity of PhyRC001 at higher concentrations (Table 2). Among them, Al^3+^ demonstrated a more pronounced inhibitory effect at all concentrations evaluated. The inhibitory effect of Mg^2+^, Cu^2+^, Fe^2+^, Mn^2+^, Ca^2+^ and Co^2+^ ions showed a direct increase in the concentration of these ions.

### 2.5. Protein Modeling 

**Figure 6:**
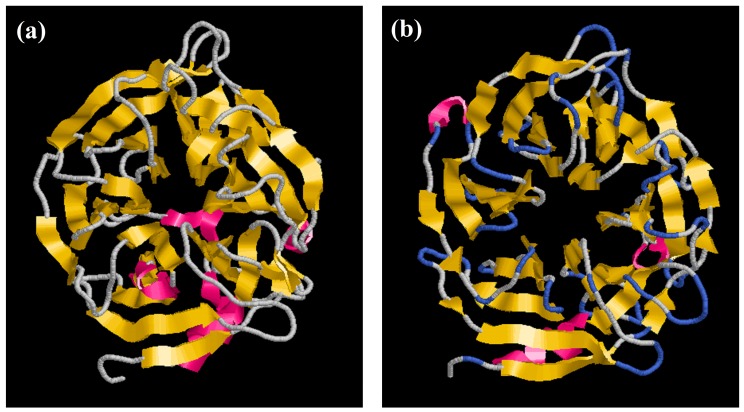
Homology modeling of phytase enzyme PhyRC001. (**a**) *B. subtilis* phytase model (3AMR, chain A); and (**b**) PhyRC001 phytase model.

Here, we report the structure of PhyRC001 (phytase). The initial structure was obtained by Comparative/Homology Modeling, performed using the MODELLER 9.13 program [18,19]. Each asymmetric unit of the structure unit cell contains one phytase monomer. The refined model contains one continuous polypeptide chain starting at residue 4 and terminating at residue 376. PhyRC001 shares 37% sequence identity with that of *Bacillus subtilis* (Figure 6a). The overall phytase complex model, just like the *B. subtilis* phytase model solved earlier, had a β-propeller consisting of five four-stranded and one five-stranded antiparallel β sheets. In the beta-sheet motif of PhyRC001, the enzyme’s active site is often found in the cleft formed in the center of the propeller by loops connecting the successive five-sheet motifs (Figure 6b).

**Figure 7:**
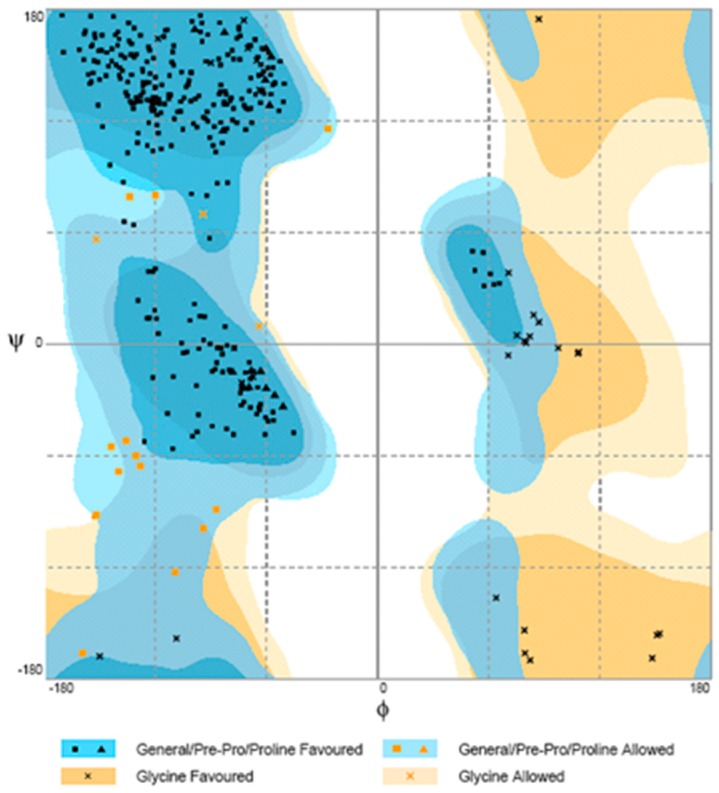
Ramachandran plot for modeled phytase obtained by PROCHECK.

The suitability of the generated model was assessed by using the general stereo chemical parameters using PROCHECK server. A Ramachandran plot of energy minimized the models of phytase structure that had been generated. The x axis of the Ramachandran plot corresponds to the Phi angles and the y axis represents Psi angles. The plots split into four quadrants which includes low energy region, allowed region, generously allowed region and disallowed region. The phytase showed 81.1% of the residues within the most favorable region, 14.9% within the moreover allowed region, 2.5% in the generously allowed region, and 1.5% in the disallowed region (Figure 7).

## 3. Discussion

New enzymatic activities have been explored through metagenomic approach in several studies, demonstrating that the diversity of environments has potential for proteins with biotechnological interest [13]. In this research, a metagenomic library was built successfully using DNA extracted from crop residues of red rice with castor bean cake, and functional screening allowed discovery of a novel phytase enzyme, which is closely related to the β-propeller phytase classes. β-propeller phytase is the only phytase that hydrolyzes phytate and liberates inorganic phosphate, when phytic acid forms an insoluble complex under neutral conditions in the presence of Ca^2+^ [20]. Therefore, because most terrestrial and aquatic environments have a neutral pH, we suggest that β-propeller phytase might be the major (and most widespread) phytate-degrading enzyme in nature and that it may play a major role in phytate–phosphorus cycling. The same hypothesis has been proposed by Lim et al. [21] based on an analysis of all the microbial and environmental sequence databases available online.

The metagenomic approach employed in this study could also be employed for the isolation of 3-phytase (EC 3.1.3.8), which can release hydrolyzes phytate and liberates inorganic phosphate. The PhyRC library was cloned and hosted in *Escherichia coli*, which allowed functional characterization of transgenically expressed enzymes and of the purified protein extracts. The molecular mass of phytases can vary over a wide size range. However, the molecular masses of the majority of previously reported phytases are from 40–100 kDa [22]. Phytases from *Aspergillus* sp., *Candida krusei* and *Schwanniomyces castellii* were reported to have a larger molecular mass: 214 kDa (a homohexamer) in *Aspergillus terreus*, 200 kDa in *Aspergillus niger*, 330 kDa in *C. krusei* and 490 kDa in *S. castellii* (a tetramer) [23]. The PhyRC001 protein purified in this study was about 45 kDa in mass, consistent with the size of proteins found in this group, which range from 38–500 kDa [23].

PhyRC001 was active over a wide pH range, maintaining 90% of its optimum activity at pH 7.0 and was stable between pH 4.0 to 8.0. On the basis of substrate specificity, two classes of phytases could be identified: phytases with broad substrate specificity and phytases which are specific for phytic acid. The phytases with broad substrate specificity exhibit significant levels of activity with a range of phosphate compounds such as β-glycerophosphate, *p*-nitrophenylphosphate and D-fructose phosphates, and degrade phytic acid to myo-inositol monophosphate or myo-inositol. In contrast, the phytases with narrow substrate specific activity are specific for myo-inositol phosphates, exhibit much lower levels of activity with myo-inositol 1-monophosphate, and result in myo-inositol tris- and bisphosphate accumulation during phytate degradation. PhyRC001 phytases belong to the latter category because phytases have high specific activity. The substrate specificity of the PhyRC001 on several phosphates was tested in 0.1 M Tris-HCl buffer (pH 7.0). Controls were included for determining initial phosphorus in each substrate. As summarized in Table 1, the enzyme had high activity for Na-IHP, but no activity on other phosphorylated compounds including sodium para-nitrophenyl phosphate, a general substrate for acid phosphatase. These results imply that the PhyRC001 is specific for inositol polyphosphate.

The need for a host to promote heterologous expression and the inability to recognize regulatory elements and the presence of different codon biases are some of the difficulties that limit success of functional metagenomics. The overall phylogenetic distribution of genes Actinobacteria (46.1%), Proteobacteria (16.7%), Firmicutes (14.2%), Chloroflexi (7.7%), and Bacteroides (6.1%) in rice straw compost enriched with manure and a microbial community involved in the diverse processes of decomposition were evaluated [24]. Although the codons from the Shine-Dalgarno promoting regions of actinobacteria differ from those of *E. coli*, thus diminishing the probability of heterologous expression, the gene we have found in the culture remains of red rice and castor bean cake was expressed in this model. We were one order of magnitude more efficient and only had to screen 50,000 clones to find one target gene. Our relatively high success rate could be, in part, explained by the wealth of phytase gene diversity or the large Gram-negative bacterial population in red rice crop residues and castor bean cake.

To facilitate the identification of phytasic activity in petri dishes, a bacteriophage lambda expression system was used, facilitating the release of the phytases expressed after lysis of *E. coli* host cells. Indeed, further efforts to purify cloned phytases heterologously expressed in *E. coli* were successful despite residual phytasic activity that was detected in cellular extracts by more sensitive colorimetric assays.

Not only functional mining of metagenomic libraries will provide the discovery of novel enzymes, but combined with other screening strategies including sequence-driven screening and high-throughput sequencing, insights into enzymatic hierarchy structure and catalytic mechanisms in specific environmental niches will be possible, as suggested by the present results.

The molecular homology structure of PhyRC001 revealed a six-bladed β-propeller in which each blade consists of a four-stranded antiparallel β-sheet (Figure 6B). β-Propeller phytases, also known as alkaline phytases, have been considered mainly identified in Bacillus species. More recent bioinformatics studies conducted on microbial genomes and environmental metagenomes suggested that the β-propeller phytases are distributed more widely than previously believed and may play a role in phytate–phosphorus cycling in soil and aquatic environment [25].

## 4. Materials and Methods 

### 4.1. Extraction and Purification of DNA from Environmental Samples

Cultural remains of red rice (leaves, steam, spikelets and straw in the ratio of 15:10:6:5 (wt. %)) and castor bean cake were submitted to temperatures from 55 to 70 °C and humidity from 80 to 90% in composting cells for 60 days at de Universidade Estadual da Paraíba (Campina Grande, Brazil). For the DNA extraction the direct lysis method [26] was used with minor modifications.

Briefly, 1 g of vegetable sample was shaken and vortexed in 2.6 mL of extraction buffer (100 mM Tris-HCl at 8.0 pH; 100 mM sodium EDTA at 8.0 pH; 100 mM sodium phosphate at 8.0 pH; 1.5 M NaCl, 1% hexadecyltrimethylammonium bromide (CTAB)). Three freeze–defrosting cycle were performed in liquid nitrogen and put at 65 °C in a water bath. After the addition of 50 µL of proteinase K (20 mg/mL), the samples were incubated at 37 °C for 30 minutes with continuous shaking at 120 rpm. Subsequently, 300 µL of 20% (*w*/*v*) SDS was added and incubated at 65 °C for 2 h with gentle shaking every 15–20 min. The supernatants were collected after centrifugation at 4000× *g* for 10 min and the resulting pellets were submitted to a re-extraction in 2 mL of extraction buffer, at 65 °C for 10 min. The combined supernatants were then admixed with 1/10 volume of 10% (*w*/*v*) CTAB [27] and centrifuged again. The resulting supernatants were extracted with chloroform-isoamyl alcohol (24:1, *v*/*v*), and the DNA was precipitated with isopropanol, washed with 70% (*v*/*v*) ethanol, dried and resuspended in 100 µL of 10 mM Tris-HCl at 8.5 pH. For DNA purification, crude DNA extracts were purified by size exclusion chromatography with CHROMA SPIN+TE-100 (BD Biosciences Clontech, Heidelberg, Germany) columns, equilibrated in 10 mM Tris-HCl at 8.5 pH, according to the manufacturer’s recommendations.

### 4.2. Construction of Genomic DNA Library and Screening

A metagenomic library was constructed using the pWEB::TNC Cosmid Cloning Kit (Epicenter, Madison, Wl, USA) according to the manufacturer’s instructions. The purified DNA had its ends repaired with T4 DNA polymerase and T4 polynucleotide kinase to generate blunt ends. For this purpose, 1.5 μg of metagenomic DNA, 1× NEB2 buffer (Biolabs), 0.5 mM dNTPs, 2 U of T4 DNA Polymerase enzyme (Biolabs) and ultrapure water were added to the final volume of 80 μL. The reaction was maintained at 12 °C for 30 min and soon after at 75 °C for 20 min in a thermocycler for inactivation of the enzyme. In the phosphorylation reaction, 1× kinase buffer (Biolabs), 2 mM ATP, 10 U T4 Polynucleotide kinase (Biolabs) and ultrapure water were added to the same tube from the previous reaction to the final volume of 90 μL. The reaction was incubated for 10 min at 37 °C and, for inactivation of the enzyme, it was kept at 65 °C for another 10 min. The DNA with the repaired ends was separated on agarose gel. DNA fragments between 20 and 60 kb were recovered from the gel and ligated to a cosmid vector pWEB::TNC which was linearized at the single *Sma*l site and dephosphorylated. Bound products (pPhytRCN01) were packaged and *E. coli* EPI100 were infected. Colonies from the library, called PhyRC, were screened for phytasic activity according to described protocols [28]. The positive clone for phytasic activity (PhyRC001) was sequenced.

### 4.3. Sequence and Phylogenetic Analyses

The possible open reading frames (ORFs) were identified with the ORF Finder at the National Center for Biotechnology Information (NCBI; http://www.ncbi.nlm.nih.gov). Closely related candidate sequences in phytase databases were identified with BlastN and BlastP by NCBI (Bethesda, MD, USA). The cloned phytase sequence and its highest scores were selected from BLAST analyses (NCBI, Bethesda, MD, USA), as well as some phytases selected from β-propeller phytases classes were also aligned for phylogenetic analyses. The histidine acid phosphatases (HAPs) phytase of *Yersinia mollaretii* ATCC 43969 (JF911533.1) was included as an outgroup. The phylogenetic tree was generated with ClustalW 1.81 [29] and MEGA 6.0 [30] using the neighbor-joining method.

### 4.4. Expression and Purification of Recombinant Phytase

The PhyRC001 sequence, excluding the sequence encoding the N-terminal signal peptide, was amplified by Polymerase Chain Reaction (PCR) using pPhytRCN01 as template and the following primers: forward primer 5′-ATAGCATGCCCGCAGCTGCGAAGATCC-3′ (containing a *Sph*I site at the 5′ end) and reverse primer 5′-ATAGTCGACCTACTCCCGCAATGCCGC-3′ (containing an *Sal*I site at the 5′ end). The amplified DNA was digested with *Sph*I and *Sal*I prior to biding to the pQE31 vector digested with the same enzymes (Qiagen, Valencia, CA, USA), resulting in the plasmid pPhytRC01ex. The recombinant plasmid, pPhytRC01ex, was the used to transform *E. coli* M15 (Qiagen, Hilden, NRW, Germany). His-tagged PhyRC001 was expressed and purified using nickel-nitrilotriacetic acid (Ni-NTA) agarose resin (Qiagen) according to the manufacturer’s instruction. Protein concentrations was determined by the Bradford method [31] using an assay kit for the quantification of protein (Bio-Rad, Hercules, CA, USA). A standard curve of protein concentration was created using known concentrations of bovine serum albumin. A Sodium Dodecyl Sulfate (SDS) – polyacrylamide gel electrophoresis (SDS-PAGE) gel analysis [32] was performed to detect the presence of expressed and purified phytase.

### 4.5. Na-IHP-Zymogram Analysis

Sodium dodecyl sulfate (SDS) polyacrylamide gel electrophoresis (SDS-PAGE) [30] was performed with some modifications to detect phytasic activity using zymogram, native-PAGE [33]. 

For the Na-IHP zymogram, an SDS-PAGE 12% gel was prepared containing 0.4% Na-IHP. The enzyme extracts were put into half of the gel at a concentration of 40 μg protein per line. This pattern was repeated to entrain the other half of the gel so that it could be cut vertically in half after electrophoresis to produce two identical acrylamide gels. The first half of the gel was stained with colloidal blue stain for visualization of proteins. The second half was used for zymogram analysis. For zymogram analysis, the native gel was incubated at 35 °C for 16 h in Na-IHP (Sigma-Aldrich, St. Louis, MO, USA) solution prepared in activity sodium acetate buffer. Activity bands were visualized by immersing the gel in a 2% (*w*/*v*) aqueous cobalt chloride solution. After a 5 min incubation at room temperature, the cobalt chloride solution was replaced with a freshly prepared solution containing equal volumes of a 6.25% (*w*/*v*) aqueous ammonium molybdate solution and 0.42% (*w*/*v*) ammonium vanadate solution. 

Phytase activity was evident as zones of clearing in an opaque background. The gel was imaged under ultraviolet light and aligned with colloidal blue stained gels.

### 4.6. Biochemical Characterization of Phytase PhyRC001

The optimum temperature for PhyRC001 activity was determined by measuring phytase activity with 1% Na-IHP (*w*/*v*) in 50 mM sodium acetate buffer, 5.0 pH, from 25 to 70 °C in increments of 5 degrees. Thermostability was examined by incubating the enzyme from 30 to 70 °C in 10 degrees increments over 60 min. The residual enzyme activity was, again, determined with 1% (*w*/*v*) phytate in 50 mM sodium acetate buffer, 6.0 pH, at 65 °C. 

Likewise, the optimum pH for enzymatic activity of PhytRC01 was determined by measuring enzyme activity with 1% (*w*/*v*) Na-IHP in 50 mM buffers, pH values ranging from 4.0 to 8.0, at 35 °C. The sodium acetate buffer was used in the pH range of 4.0 to 6.0. Sodium phosphate buffer was used for pH range between 6.0 and 8.0. To determine the stability of the enzymatic pH, the extract containing the enzyme was incubated at different pHs, as mentioned above, at 4 °C for 16 h. The residual enzyme activity was measured under standard assay procedure (1% phytate in 50 mM sodium acetate buffer, pH 6.0, at 35 °C).

To investigate the substrate specificity, the enzymatic activities were tested under optimal conditions for 30 min with 1% (*w*/*v*) organic phosphate such as Phytic acid (Fluka, St. Louis, MO, USA), para-Nitrophenyl phosphate (Sigma, St. Louis, MO, USA), AMP (Sigma), ADP (Sigma), ATP (Sigma), GTP (Sigma), NADP (Sigma), Glucose 1-phosphate (Sigma) and Glucose 6-phosphate (Sigma).

To inspect the influence of metal ions, the enzymatic activities were tested under optimal conditions for 30 min with (1, 5, 10 and 50 mM) metal ions such as Ca^2+^ (Sigma), Mg^2+^ (Sigma), Al^3+^ (Sigma), Cu^2+^ (Sigma), Zn^2+^ (Sigma), Fe^2+^ (Sigma), Ni^2+^ (Sigma), Mn^2+^ (Sigma) and Co^2+^ (Sigma).

### 4.7. Protein Modeling of Phytase PhyRC001

The homology molecular modeling methodology involved four successive steps: identification and selection of the template protein; aligning the target and template sequences; construction and optimization of the model and validation of the model.

The amino acid sequence of the PhyRC001 protein was subjected to a comparative analysis through the BLAST (Basic Local Alignment Search Tool, NCBI, Bethesda, MD, USA) program (www.ncbi.nih.gov/BLAST) [34] in the PDB database. The amino acid sequences from some of the microorganisms found in the search result were submitted to the multiple sequence alignment program (CLUSTALX). In this first step, a protein related to the amino acid sequence of PhyRC001 (target protein) was identified, taking into account during the process of choosing the template protein, aspects such as: structural knowledge, sequence similarity, function similarity, expression by the same group of genes and evolutionary correlation between the proteins, 3AMR: chain A [35].

Identified to the protein template, overall alignment methods between the sequences were applied, which consisted of the second step of comparative modeling. The alignment between sequences was performed using the CLUSTALW program (http://www2.ebi.ac.uk/clustalw/). The aim of this alignment was to recognize structurally conserved regions and variable regions, observing the structurally equivalent residues in the primary sequence.

The third step of the modeling was the construction of the model, which was based on the information contained in the alignment generated between the sequences. The model was constructed using the MODELLER program [18]. The modeling used satisfied all spatial constraints, using geometric distances and optimization techniques. The visualization of the three-dimensional structure was performed with the RASMOL program [36].

The quality of the generated 3D model was validated by the Ramachandran plot [37] with the PROCHECK program [38], which evaluated the three-dimensional structure of the protein indicating its stereochemical quality. The quality of the model was linked to the choice of the mold and the construction of the alignment that serves as a reference for the construction of the atoms that compose the protein.

## 5. Conclusions

In conclusion, an enzyme belonging to the β-propeller phytase family was isolated successfully using a functional approach to screen a metagenomic library built from DNA isolated from red rice crop residues with castor bean cake. The screen involved looking for phytasic activity in a heterologous expression system in *E. coli* PhyRC001 and subsequent purification and characterization of the recombinant protein.

## Figures and Tables

**Table 1:** Substrate specificity of PhyRC001, shown by the relative activity of PhyRC001 for different substrates.

Substrate	Relative Activity (%)
Phytic acid	100
AMP	0
ADP	0
ATP	0
GTP	0
NADP	0
para-Nitrophenyl phosphate	19
Glucose 1-phosphate	1
Glucose 6-phosphate	1

**Table 2:** Effects of metal ions on the relative phytase activity of PhyRC001.

Relative Phytase Activity,%				
Metal Ions	1 mM	5 mM	10 mM	50 mM
Ca^2+^	100	100	98	75
Mg^2+^	96	73	23	5
Al^3+^	6	0	0	0
Cu^2+^	80	66	13	0
Co^2+^	88	66	40	7
Mn^2+^	100	95	45	15
Zn^2+^	87	90	35	0
Fe^2+^	60	30	10	n/a*
Ni^2+^	55	60	20	10

n/a*: Fe^2+^ at high concentration interferes with the colorimetric assay reagents. The phytase activity was measured at pH 7.0, 35 °C, and is expressed as a percentage (average of three replicates) of the activity level in the absence of the metal ions. For all the metal ions in the table, the anion of the chemical used is Cl^−^. Relative average standard deviation (±1.24).
